# Endosomal Interactions during Root Hair Growth

**DOI:** 10.3389/fpls.2015.01262

**Published:** 2016-01-29

**Authors:** Daniel von Wangenheim, Amparo Rosero, George Komis, Olga Šamajová, Miroslav Ovečka, Boris Voigt, Jozef Šamaj

**Affiliations:** ^1^Department of Plant Cell Biology, Institute of Cellular and Molecular Botany, University of BonnBonn, Germany; ^2^Department of Cell Biology, Faculty of Science, Centre of the Region Haná for Biotechnological and Agricultural Research, Palacký UniversityOlomouc, Czech Republic

**Keywords:** endosomes, trafficking, interaction, *Arabidopsis thaliana*, root hair, development, spinning disc microscopy, structured illumination microscopy

## Abstract

The dynamic localization of endosomal compartments labeled with targeted fluorescent protein tags is routinely followed by time lapse fluorescence microscopy approaches and single particle tracking algorithms. In this way trajectories of individual endosomes can be mapped and linked to physiological processes as cell growth. However, other aspects of dynamic behavior including endosomal interactions are difficult to follow in this manner. Therefore, we characterized the localization and dynamic properties of early and late endosomes throughout the entire course of root hair formation by means of spinning disc time lapse imaging and post-acquisition automated multitracking and quantitative analysis. Our results show differential motile behavior of early and late endosomes and interactions of late endosomes that may be specified to particular root hair domains. Detailed data analysis revealed a particular transient interaction between late endosomes—termed herein as dancing-endosomes—which is not concluding to vesicular fusion. Endosomes preferentially located in the root hair tip interacted as dancing-endosomes and traveled short distances during this interaction. Finally, sizes of early and late endosomes were addressed by means of super-resolution structured illumination microscopy (SIM) to corroborate measurements on the spinning disc. This is a first study providing quantitative microscopic data on dynamic spatio-temporal interactions of endosomes during root hair tip growth.

## Introduction

The eukaryotic endomembrane system plays an essential role in the synthesis, sorting, delivery, storage, recycling, and degradation of macromolecules within the cell (Contento and Bassham, 2012; Hao et al., 2014). Endosomes are organelles involved in sorting, signaling and selective cargo degradation within the endomembrane system (Šamaj, 2012). During the last decade, early and late endosomes were identified and partially functionally characterized in plants (Dhonukshe et al., 2007; Müller et al., 2007; Chen et al., 2011; Reyes et al., 2011; Sharfman et al., 2011; Fan et al., 2013). Early endosome/*trans*-Golgi network (TGN) represents a subcellular organelle merging endocytotic and secretory pathways (Dettmer et al., 2006; Chow et al., 2008; Viotti et al., 2010; Contento and Bassham, 2012; Qi and Zheng, 2013). The late endosomes/multivesicular bodies mature from TGN/early endosomes (Scheuring et al., 2011; Park and Jürgens, 2012) and are involved in the biosynthetic or degradative transport to the vacuole (Bottanelli et al., 2011; Contento and Bassham, 2012).

Endosomes are essential for developmental processes in plants including tip growth of root hairs (Voigt et al., 2005a) and pollen tubes (Richter et al., 2011; Idilli et al., 2013), cell plate formation during cytokinesis (Dhonukshe et al., 2006), plant immunity (Spallek et al., 2013), auxin distribution (Ischebeck et al., 2013), and organ development (Li et al., 2012b; Kim et al., 2013). Nevertheless, very little is known about dynamic aspects of endosomal behavior such as their interactions, speed, and movement patterns in plant cells, particularly in highly polarized cells such as root hairs.

Root hairs are polar tubular outgrowths of trichoblasts (root hair-forming cells) which elongate exclusively at their tips. Since mode of growth is determined by well-organized vesicular trafficking interconnected with cytoskeleton dynamics they represent an ideal model to study tip growth (Ovečka et al., 2005; Voigt et al., 2005a,b; Šamaj et al., 2006). Root hair initiation is coupled to actin cytoskeleton rearrangements (Ringli et al., 2002), modification of the cell wall composition (Park et al., 2011), and accumulation of structural sterols in the plasma membrane (Ovečka et al., 2010). These local structural changes may regulate the bulge formation and subsequently, the root hair elongation by modulation of vesicular trafficking.

In growing root hairs, the tip growth requires an adequate balance of macromolecules supply, retrieval and recycling. For this reason, the apical zone is filled with secretory and endocytotic vesicles (Cole and Fowler, 2006; Campanoni and Blatt, 2007). Rapid endocytosis was detected by internalization and transport of FM dyes through highly dynamic early endosomes in the clear zone to larger endosomal compartments in the subapical region (Ovečka et al., 2005).

By means of fluorescence microscopy, several proteins have been found to be localized at distinct endosomal compartments. RabA1d, a member of the RabA1 subfamily of small GTPases, and VTI12, a SNARE-protein, accumulate at the early endosome/TGN compartments (Sanderfoot et al., 2001; Uemura et al., 2004; Ovečka et al., 2010; Berson et al., 2014). Similarly, other Rab-GTPases are involved in endocytotic processes but they are localized preferentially in late endosomes, such as RabF2a, RabF2b, and RabF1 (Ueda et al., 2004; Haas et al., 2007; Geldner et al., 2009). FYVE domain marker binds specifically to the phosphoinositol-3-phosphate (PI-3P) mainly in the membranes of late endosomal compartments (Gillooly et al., 2001) and colocalizes with RabF2a and RabF1 (Ueda et al., 2004; Voigt et al., 2005a; Haas et al., 2007).

The endosomal compartments distributed along the root hairs showed different patterns of motility, including stationary phases, slow, or rapid movements (Voigt et al., 2005a). This motility is influenced by both actin and microtubule cytoskeleton (Voigt et al., 2005a; Idilli et al., 2013) and their motor proteins, i.e., myosins (Peremyslov et al., 2012). In functional analysis of endosomal dynamics and regulation in mammalian cells (Gasman et al., 2003; Lai et al., 2009; Flores-Rodriguez et al., 2011; Ohashi et al., 2011) and yeast (Toshima et al., 2006), automatic and semi-automatic tracking has been used to obtain parameters such as endosome size, organization, and motility. However, contrary to mammalian cells and yeast, the organization and dynamic behavior of plant endosomes have not been investigated extensively.

Advanced microscopy together with automatic single-particle tracking (SPT) techniques are useful to gain new important information about mechanisms and structures in living cells (Sbalzarini and Koumoutsakos, 2005; Flores-Rodriguez et al., 2011; Ruthardt et al., 2011). Accurate tracking and quantitative analysis of endosomal identification, selection, and trajectory calculation require appropriate algorithms (Sbalzarini and Koumoutsakos, 2005). Different programs for tracking of endosomes and vesicles have been used, such as Motion Track program (Gasman et al., 2003), PolyParticleTracker (Flores-Rodriguez et al., 2011), ImageJ (Toshima et al., 2006; Ohashi et al., 2011; Li et al., 2012a), or DiaTrack (Trejo et al., 2010), which use individual algorithms to recognize and follow particles.

In this study, the localization, dynamic properties, and interactions of endosomes through the entire course of root hair formation were quantitatively characterized using time-lapse live imaging coupled to multitracking analysis. Analyses at high temporal and spatial resolution revealed differences in organization and mobility between early and late endosomes in diverse root hair growth stages. Late endosomes showed temporal interactions and clustering/fusion events that were recognized and quantified at certain stages of root hair growth and development. A new type of transient interaction of late endosomes, called dancing-endosomes was described in more detail.

## Materials and methods

### Plant material

Arabidopsis seedlings expressing early and late endosomal fluorescently-tagged proteins were used. Early endosomes were visualized by YFP-VTI12 and GFP-RabA1d (Sanderfoot et al., 2001; Uemura et al., 2004; Ovečka et al., 2010; Berson et al., 2014), while late endosomes were visualized by YFP-RabF2a, YFP-RabF2b, and GFP-2xFYVE tagged proteins (Gillooly et al., 2001; Ueda et al., 2004; Voigt et al., 2005a; Haas et al., 2007; Geldner et al., 2009). Seeds were sown on 1/2 MS plates and cultured vertically for 5–7 days under long day conditions (16 h light/8 h darkness period with 120–140 μmol m^−2^ s^−1^ light, at 22°C).

Before imaging, plants were transferred for adaptation to microchambers (containing three layers of Parafilm as spacer between the microscope slide and the coverslip) filled with liquid 1/2 MS medium (Ovečka et al., 2005). Microchambers with plants were placed in a sterile staining cuvette which contained liquid 1/2 MS up to a few millimeters higher that the lower edge of the microchamber and kept there for a few hours.

### Image acquisition

Fluorescence microscopy was performed with the Olympus IX71 microscope combined with a spinning disc unit and an EMCCD camera (Andor Technology, Belfast, UK) or with Zeiss Observer Z1 microscope combined with a Yokogawa CSU-X1 spinning disc unit and the high-resolution Evolve 512 black-thinned EM-CCD camera (Photometrics). GFP- and YFP-tagged constructs were excited at 488 nm and 514 nm, respectively, and fluorescence was detected between 500 and 560 nm. Images were collected using the UPlanSApo 60x/1.2 water immersion objective or Alpha Plan Apochromat 100x, NA 1.57 oil objective lenses. Images were acquired with 0.26 μm/pixel and time interval between 0.1 and 0.7 s/frame.

Endosomal size measurements were performed using the Olympus FLUOVIEW FV1000 confocal microscope. Images were collected using the UPLSAPO 60x/1.35 oil immersion objective lens and a pixel size of 0.1 μm. Images were acquired in a single plane with a sampling speed of 2 μs/pixel.

Root hair tip growth rate of Col-0 and GFP-2xFYVE lines was measured on 2-days-old seedlings after transferring them into micro-chambers filled with liquid 1/2 MS medium and stabilizing for a few hours in standard culture conditions. Growing root hairs were selected under the microscope and images were captured every 60 s for a time period of 20 min with Zeiss FC PlanNeofuar 40x/0.75 objective and Zeiss AxioCam ICm1 camera. Data were analyzed using Zeiss Zen Blue 2014 software. In total 24–33 growing root hairs from three individual plants per line were analyzed.

### Morphological and dynamic estimations by automatic analysis

We used multitracking software DiaTrack 3.02 (Vallotton Semasopht Corp., Chavannes-pres-Renens, Switzerland) for simultaneous multiple tracking and quantification analyses of endosomal dynamics (Vallotton and Olivier, 2013). The program allows analyzing several parameters including size, speed, movement, shape, etc. DiaTrack was used to estimate and compare the diameter of the different endosomal compartments. The size measurement in DiaTrack is based on a watershed transformation and a size measurement using the function regionprops in MATLAB. Endosome diameter was measured in each frame of the image sequence and compartments were automatically measured several times to confirm the results which were presented as frequency of endosomes in the cell. Apical regions were defined as the first 10 μm below the tip and subapical as the rest of the root hair area. In DiaTrack, trajectories of individual endosomes were tracked according to their properties, e.g., a gray value threshold excludes objects in the background noise. Furthermore, trajectories shorter than 5 μm were excluded. The ImageJ plugin MultipleKymograph was used to generate kymographs for qualitative analysis of movement patterns of the endosomal compartments and quantitative measurement of their speed. Kymographs were obtained by drawing a line (1 px width) along a cytoplasmic strand, subapical at least 10 μm below the tip. In kymographs, only straight lines (showing constant speed of endosomes) that were at least 15–20 μm long were assigned as a continuous movement. The maximum speed of endosomal movement was calculated from the steepest slope of the resulting kymographs.

### Structured illumination microscopy

SIM images were acquired with an Elyra S.1 platform (Zeiss, Munich, Germany) using a 100 ×/NA 1.57 Plan Apochromat objective according to previously published work (Komis et al., 2014, 2015). For optimal superresolution of relatively immotile endosomes, images were formed by deconvolving Moiré patterns resulting from the sample and a 34 μm physical grating (the final optical pattern has a period of ca. λ_*exc*_/2 nm) that was rotated 5 times (at 72° increments) and phase shifted 5 times (at 2π/5 increments) per rotation. For time lapse imaging, rotations were reduced to three (at 120° increments) without changing the phase shifting. Images were captured on a pco.edge 5.5 sCMOS camera (PCO, Kelheim, Germany), 2560 × 2160 pixels, pixel size 6.5 × 6.5μm, readout noise < 1.0 at 30 fps and < 1.5 at 100 fps, 16 bit dynamic range and peak quantum efficiency >60%. Calculation of the super-resolution images was done with Zen 2014 software (Black version) with built in SR SIM plugin. Raw and reconstructed images were validated in Fourier space according to standard procedures, again using the Fast Fourier Transform of Zen 2014. To approximate resolved endosome sizes, we used FWHM of linear normalized intensity profiles drawn across individual endosomes using previously published procedure (Komis et al., 2014, 2015).

### Area fraction analysis

Abundance of endosomes in root hairs was estimated by determination of area fraction. Maximum z-projection images at one time point were produced from xyzt series after standard thresholding (standardized settings for all images). The area fraction occupied by the fluorescent signal of tagged-proteins (in pixels) in apical and subapical regions was determined using ImageJ function. Three replicates were analyzed.

### Detection and quantification of endosomal interactions and fusions

One z-frame was selected from acquired xyzt-series (4-D imaging) in order to ensure a specific endosomal population and to avoid overlapping structures from different z-frame. The selected 8 bits time-lapse series were analyzed using TrackMate function in ImageJ. The segmentation procedure was done by subtracting two consecutive Gaussian convolutions using Difference of Gaussian (DoG) detector, which efficiently detects small particles. The settings were established for all series as follows: diameter 1.5 px, threshold 2, using median filter and sub-pixel localization (Schindelin et al., 2012). Endosome population was determined as number of spots identified in the first frame in bulge stage and in tip zone of root hairs (tip-ROI of 30 μm length). Total intensity was viewed in color-coded scale using HyperStack displayer and subsequently, the identified spots were visually followed, the occurred events were classified and counted. The physical interaction during dancing-endosomes was determined by nearby location (less than 1 μm, defined manually by subpixel estimation) or temporal intensity increase. The increase in the fluorescence intensity which remained along the observation time suggested a size increase and thus, it was recognized as clustering or fusion event (Helmuth et al., 2009; Puchner et al., 2013). Six image series of bulge stage and mature root hair, and 24 of growing root hairs were used to define the endosomal population. The speed and distances in individual, interacting, and clustered endosomes were measured in growing root hairs using the semi-automatic MtrackJ function in ImageJ (Meijering et al., 2012). Endosomes were selected and speed, distance, and intensity were determined as parameters during structure displacement. Automatic comparison of all points of the track helped to obtain maximal, mean, and minimum values per trajectory for each parameter (speed, distance, and intensity). More than 20 endosomes from eight growing root hairs were randomly selected, classified as individual, interacting, or clustered and measured to estimate speed and distances.

## Results

### Size and intracellular distribution of endosomal compartments during root hair development

**Figure 1:**
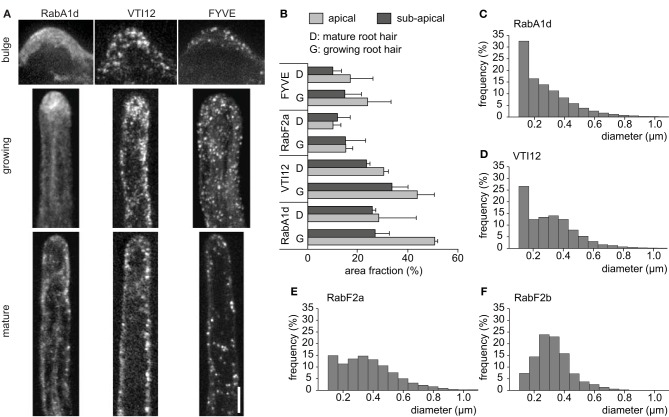
**Localization and size of different endosomal compartments during root hair growth**. **(A)** Early endosomes marked by GFP-RabA1d and YFP-VTI12, and late endosomes marked by GFP-2xFYVE during root hair development, in the bulge, growing, and mature root hair. Maximum intensity projections from z-stacks composed of 5–8 optical sections are shown. **(B)** Area occupied by early and late endosomes in growing and mature root hairs. The area fraction occupied by the fluorescent signal of fluorescently tagged-endosomal markers (in pixels) in apical and subapical regions of root hairs was determined using ImageJ function from three replicates. **(C–F)** Distributions of early and late endosomes according to size measurements. Image sequences from four to six root hairs from individual plants were analyzed for each endosomal compartment. Scale bar represents 10 μm. See also Supplementary Movie S1.

Endosomal compartments showed different intracellular distributions in emerging and developing root hairs (Figure 1A). During bulge formation in root trichoblasts, the early endosomes visualized with GFP-RabA1d and YFP-VTI12 markers accumulated at the tip of the emerging root hair, while the late endosomes visualized by GFP-2xFYVE marker were scarce. The fast growing root hairs showed a characteristic cytoplasmic streaming known as “reverse-fountain-like pattern” (Ovečka et al., 2005, 2010). In growing root hairs, the early endosomes visualized by GFP-RabA1d and YFP-VTI12 markers showed a tip-accumulation, whereas the late endosomes visualized by GFP-2xFYVE marker showed a more homogeneous distribution throughout the entire root hair shank. No obvious accumulation of early endosomes or late endosomes at the tip was observed in mature root hairs (Figure 1A; Supplementary Movie S1). This observation was also confirmed by determination of area fraction in apical and subapical regions showing that the early endosomes visualized with GFP-RabA1d and YFP-VTI12 markers had significantly higher presence in the apical region of growing root hairs than the late endosomes visualized by YFP-RabF2a and GFP-2xFYVE markers (Figure 1B). In general, the growing root hairs showed higher area fraction of both early and late endosomes than mature root hairs.

DiaTrack software (Vallotton and Olivier, 2013) was used to automatically identify and estimate the endosome diameter for the different endosomal populations (Materials and Methods, Supplementary Figures S1A,B; Supplementary Table S1). Early and late endosomes showed significant differences with respect to their diameter (Figures 1C–F). Early endosomes visualized by GFP-RabA1d and YFP-VTI12 markers showed higher frequency at lower diameter (Figures 1C,D) compared to the late endosomes visualized by YFP-RabF2a and YFP-RabF2b markers (Figures 1E,F). The size distribution of individual endosomal compartments suggests that they appear in different sizes, e.g., as single endosomes or clusters. The distribution, accumulation and size pattern of early and late endosomes appeared to be specific to different stages of root hair growth (Figures 1A,B).

**Figure 2:**
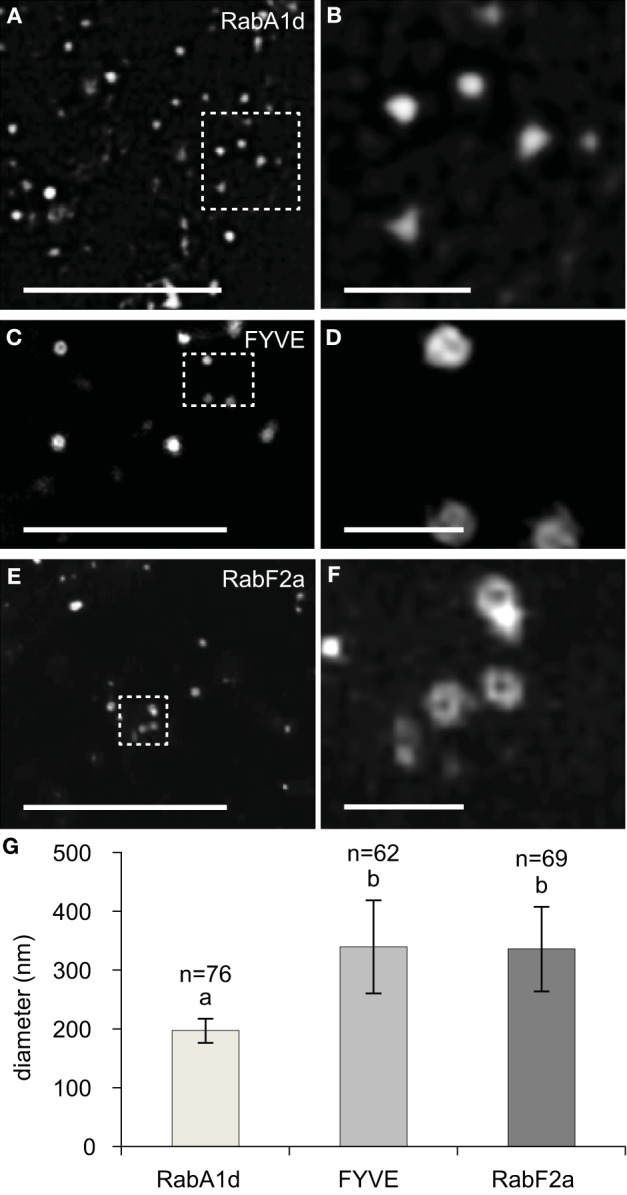
**Overview, detailed imaging, and quantification of early and late endosomal size followed by SIM**. **(A,B)** Overview **(A)** and details (**B**, corresponding to boxed area of **A**) of early endosomes labeled with GFP-RabA1d. Early endosomes appear as bright fluorescent spots. **(C,D)** Overview **(C)** and details **(D)** of vesicles labeled with GFP-2xFYVE late endosomal marker. **(E,F)** Overview **(E)** and details **(F)** of YFP-RabF2a labeled late endosomes, showing the same hollow structure as the GFP-2xFYVE labeled ones. **(G)** Graphic depiction of average early and late endosomal diameters labeled with GFP-RabA1d, GFP-2xFYVE, and YFP-RabF2a, respectively. Different letters above the bars indicate significant difference (Student *t*-test, *p* < 0.001). More than 62 endosomes from over 20 cells of at least five individual plants were analyzed for each endosomal compartment. Scale bars: 10 μm **(A,C,E)**; 1 μm **(B,D,F)**.

All of the size distributions of GFP-RabA1d labeled early endosomes as well as YFP-RabF2a or GFP-2xFYVE labeled late endosomes were approximated from images in the spinning disc microscope which is diffraction limited and cannot relate to their actual size. Therefore, sizes of endosomal compartments were further followed by SIM, providing the means to image at a lateral resolution threshold at ca. 100 nm (Komis et al., 2014, 2015). In order to address sizes of imaged endosomal structures we generated an indicative dataset of early and late endosome diameters obtained by SIM imaging. Individual early endosomes labeled with GFP-RabA1d were resolved as spots of variable size with Gaussian fluorescence intensity distribution around their center (Figures 2A,B). By means of intensity profiling we extrapolated full width at half maximum values (FWHM) of individual early endosomes as a measure of their resolution via SIM. Late endosomes were resolved as ring-like structures representing their vesicular form as it is externally coated by the GFP-2xFYVE and the YFP-RabF2a late endosomal markers (Figures 2C–F, respectively). According to FWHM values, early endosomes exhibited a range of diameters between 154 and 251 nm averaging at 197 ± 20.7 nm (*n* = 76; Figure 2G). In the same manner by extrapolating FWHM values of late endosomes, we deduced average diameters of 336.2 ± 72 nm and 339.7 ± 79 nm, respectively (*n* = 62 and *n* = 69, respectively) which were significantly different when compared to early endosomes (Figure 2G).

### Dynamics of endosomes in growing root hairs

**Figure 3:**
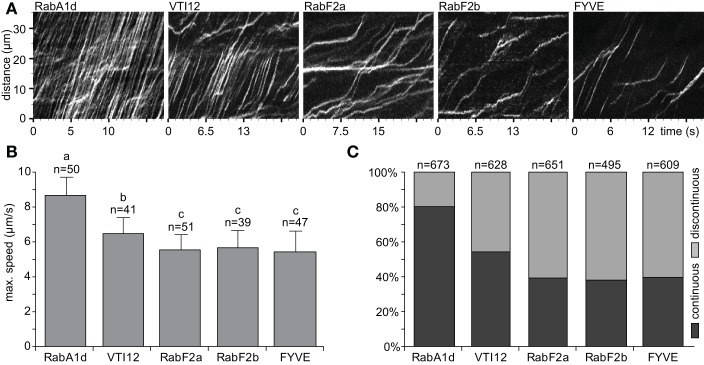
**Dynamics of early and late endosomes in root hairs**. **(A)** Kymographs, a line (width = 1px) along a cytoplasmic strand in the middle of a cell (subapical), show movements of the early endosomes visualized by GFP-RabA1d and YFP-VTI12 and late endosomes visualized by YFP-RabF2a, YFP-RabF2b, and GFP-2xFYVE. Single slice image acquisition was performed (50 ms exposure time and 10–15 frames per second). **(B)** The maximum speed of endosomes was determined in kymographs by eye (steepest slopes) in mature root hairs. More than 39 endosomes from over 20 cells of at least 10 individual plants were analyzed for each endosomal compartment. **(C)** Proportional ratio between continuous and discontinuous movements of endosomes. Non-interrupted movements along a straight line, at least 15–20 μm long, were assigned as continuous while all other types of movements were assigned as discontinuous ones. This classification was performed on the same kymographs used in **(B)**. Different letters above the bars indicate significant difference from Wilcoxon rank sum test with Holm's correction (*p* < 0.05).

Early and late endosomes showed different dynamic behavior mainly in terms of speed and trajectory. During continuous movement, endosomes moved by constant speed and followed a certain direction or trajectory. On the other hand, endosomes moving by discontinuous movements showed sudden changes either in speed or direction or both. Such continuous movements of early endosomes visualized by GFP-RabA1d and YFP-VTI12 markers were visible in respective kymographs exhibiting several parallel skewed stripes representing continuous movement with constant speed within the root hair (Figure 3A). Meanwhile, the kymographs of the late endosomes marked by YFP-RabF2a, YFP-RabF2b, and GFP-2xFYVE exhibited more irregular patterns with frequent changes in speed referred here as discontinuous movements. The maximal speed was determined from highly dynamic endosomes depicted in the kymograph (Supplementary Figures S1C,D). Early endosomes visualized by GFP-RabA1d marker showed the highest speed (8.7 ± 1.1 μm/s), followed by YFP-VTI12-labeled endosomes (6.5 ± 0.9 μm/s; Figure 3B). Late endosomes visualized by YFP-RabF2a (5.6 ± 0.9 μm/s), YFP-RabF2b (5.7 ± 1.1 μm/s), and GFP-2xFYVE (5.5 ± 1.2 μm/s) markers moved slower than early endosomes (Figure 3B).

Finally, the patterns of continuous and discontinuous movements were classified and quantified for each of the endosomal compartments (Figure 3C; Supplementary Table S2). Eighty percent of early endosomes visualized by GFP-RabA1d marker moved mainly in a continuous pattern whereas late endosomes visualized by YFP-RabF2a, YFP-RabF2b, and GFP-2xFYVE markers showed only 40% of continuous motility. Early endosomes visualized by YFP-VTI12 marker showed nearly equally continuous and discontinuous movements (Figure 3C).

### Speed alterations of late endosomes in growing root hairs

**Figure 4:**
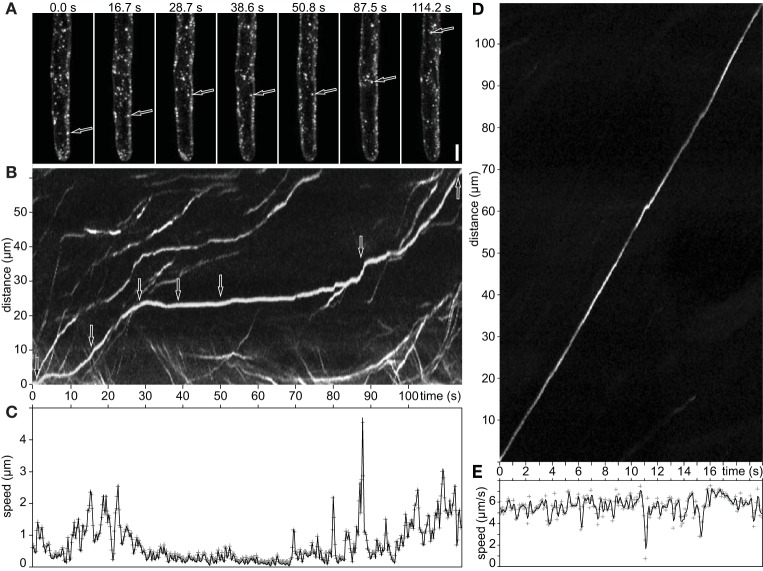
**Movement of late endosomes visualized by GFP-2xFYVE in growing root hairs**. **(A)** Maximum intensity projection of three images (3 μm z-spacing). Discontinuous stop-and-go movement of late endosomes. **(B)** Pixels along its trajectory are plotted as a function of time (kymograph). The arrows in **(A,B)** highlight one moving endosome at different time points. **(C)** Speed profile of the endosome showed in **(A,B)** measured with DiaTrack. **(D)** Kymograph documenting continuous movement of a late endosome. **(E)** Speed profile from **(D)**. Scale bar represents 10 μm. See also Supplementary Movie S2.

The discontinuous motility pattern of late endosomes was characterized by time-lapse imaging of GFP-2xFYVE labeled endosomes. Such discontinuous pattern, composed of alternating slow and fast movements, was analyzed by using particle tracking and kymographs along their trajectories (Figure 4; Supplementary Movie S2). Both, kymographs and speed measurements revealed abrupt changes of the endosomal velocity with alternating periods of fast movement with maximum speed (go phase) and immobility (stop phase). The stop phase lasted for a certain time and was observed several times during late endosome movement (Figures 4A–C; Supplementary Movie S2). The transition from “stop” to “go” phase exhibited variable speed, with high or slow accelerations (Figure 4C). In contrast, the continuous movement showed a straight line in the kymograph indicating minor changes in speed (Figures 4D,E).

### Endosomal interactions and synchronized movements

**Figure 5:**
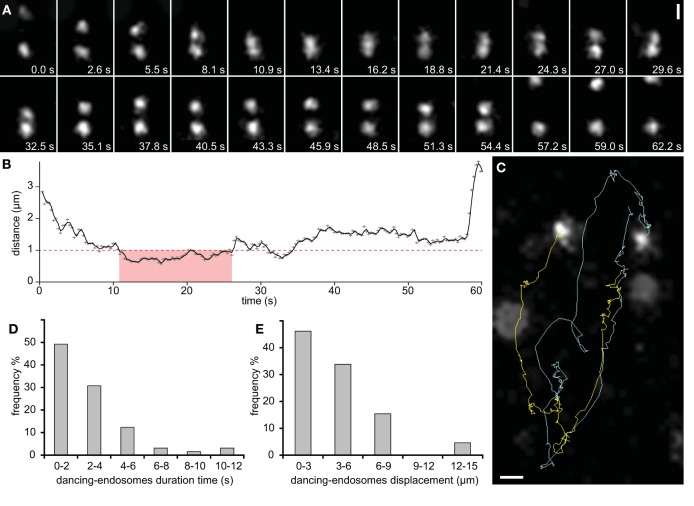
**Dancing endosomes visualized with GFP-2xFYVE**. **(A)** Two late endosomes approach each other and move together forth and back through the cell for almost 1 min before they separate from each other (observation in a single slice). **(B)** Their synchronized movement was accompanied by changes in distance over time. Physical interactions between them were recognized at closest distance, which was < 1 μm (marked by red dashed line) and within short time period (marked by pink color). **(C)** Automatic tracking of dancing-endosomes. **(D)** Frequency of dancing-endosomes with respect to the time. **(E)** Frequency of dancing-endosomes with respect to their displacement distance, recorded during their close interactions. Sixty-five endosomes from 19 growing root hairs were used for speed and distance estimations during dancing interaction of endosomes. Scale bars represent 1 μm for **(A,C)**. See also Supplementary Movies S3, S4.

Detailed observations revealed that some adjacent late endosomes visualized by GFP-2 × FYVE marker can move in a synchronized manner for a considerable time period (between 10 and 35 s) suggesting tethering of such endosomes during this synchronized movement. These endosomes approached each other and subsequently, kept a relatively constant distance for some period of time before they quickly moved away (Figures 5A,B; Supplementary Movies S3, S4). This period of time can be separated into two parts based on the distances, a very close proximity (< 1 μm from 11 to 27 s) and a distance of about 1.5 μm in seconds 25–57 (Figures 5A,B). We term this temporal interaction as dancing-endosomes. A possible physical interaction can be considered as the closest distance between endosomes residing within a distance smaller than 1 μm lasted between 10 and 12 s (Figures 5A,B). Individual trajectories of the endosomes showed a synchronized movement (Figures 5A,C; Supplementary Movies S3, S4). At the closest distances (defined manually as the distance between the centers of two spots using subpixel precision), dancing-like interactions between two late endosomes were more frequent during a brief period of time (Figure 5D) while they moved over short distances (Figure 5E). Similar pattern of synchronized interactions including common dancing was typical also for late endosomes visualized by YFP-RabF2a marker (Supplementary Movie S5).

**Figure 6:**
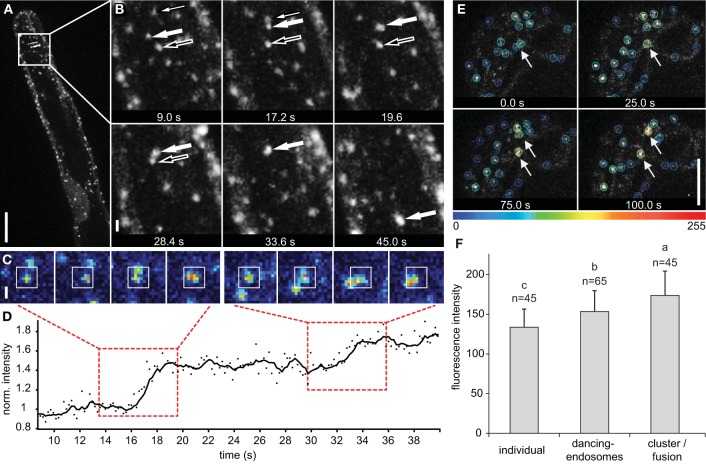
**Late endosomes visualized by GFP-2xFYVE undergoing clustering/fusion**. **(A)** Maximum intensity projection of four z-slices per time point (2 μm z-spacing) showing the process of endosomal clustering or fusion in growing root hairs. **(B)** One of the z-slices shows a detailed observation of endosomal clustering or fusion in time. Three endosomes marked by different arrows approximate to each other and sequentially cluster/fuse together producing large endosome. **(C)** Sequence of images from real-time imaging shown in **(A,B)** documenting clustering/fusion of individual late endosomes. Images are color-coded according to fluorescence intensity. **(D)** The coordinates from the cell tracking were used to measure the fluorescence intensity of interacting endosomes within a 7 × 7 pixel area (indicated by white box) shown in **(C)**. Fluorescence intensity was normalized to the initial value of one individual endosome (marked by big filled arrow in **B**) and plotted against time. Time periods of clustering shown in **(C)** are highlighted with red dashed boxes. **(E)** Detection of endosome clustering/fusion events based on increase of total fluorescence intensity. Color-coded marking of individual and clustered/fused endosomes in the bulge stage of root hair development. Increasing of the fluorescence intensity due to clustering/fusion is highlighted by arrows. **(F)** Mean fluorescence intensity of individual endosomes, dancing-endosomes and endosomes after clustering/fusion in growing root hairs. Sixty-five endosomes from 19 growing root hairs were used to determine fluorescence intensity during endosomal interactions. Error bars represent SD. Different letters above the bars indicate significant difference (Student *t*-test, *p* < 0.05). Scale bar represents 10 μm **(A,E)** and 1 μm **(B,C)**. See also Supplementary Movie S6.

In time-lapse images, the physical interaction of late endosomes was determined by nearby location or temporal intensity increase (Figures 6A–D; Supplementary Movie S6). Occasionally, this increase in the fluorescence intensity remained along longer observation time (over 20 s), suggesting clustering and/or putative fusion events between the interacting endosomes. Two late endosomes came to the close contact, clustered and/or putatively fused together and eventually moved toward another endosome following next clustering and/or putative fusion (Figures 6A,B). Consistently, a significant fluorescence increase was observed after endosome clustering and/or putative fusion occurred (Figures 6C,D). All these events were followed using a color-coded scaling according to the total fluorescence intensity (Figure 6E). The total fluorescence intensity of clustered and/or putatively fused endosomes was significantly higher than in individual and dancing-endosomes (Figure 6F).

**Figure 7:**
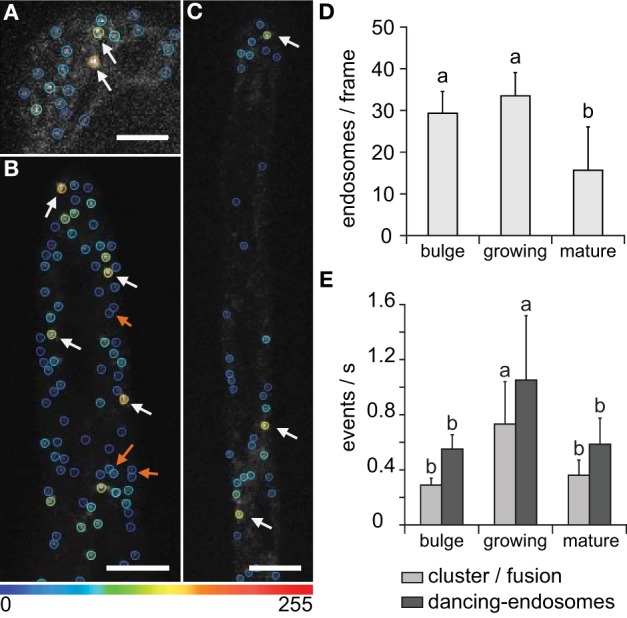
**Interactions of late endosomes visualized by GFP-2xFYVE during different phases of root hair development**. **(A–C)** Color-coded marking of individual and clustered/fused endosomes in bulge stage **(A)**, in growing root hair **(B)**, and in mature root hair **(C)**. Increase in total fluorescence intensity after clustering/fusion changed color-coding of late endosomes from blue to yellow and orange (white arrows). The physical interaction of dancing-endosomes is determined by nearby location or temporal increase in fluorescence intensity (orange arrows). All images correspond to one z-frame selected from acquired xyzt-series, color coding showing the intensity changes after 5 s of recording. **(D)** Endosomal population among different root hair developmental stages. **(E)** Quantification of dancing-endosomes and clustering/fusions of late endosomes per second in the bulge and in the apical zone (first 10 μm at root hair tip) of growing and mature root hairs. Single Z-stack time series of six root hairs at bulge stage, six of mature root hairs, and 24 of growing root hairs were used to define the endosomal population (using single image, **D**) and time series to define and follow endosomal behavior **(E)**. Error bars represent SD. Different letters above the bars indicate significant difference (Student *t*-test, *p* < 0.05). Scale bar represents 10 μm.

Comparative observations of individual, dancing-, and clustered endosomes during bulge formation in root trichoblasts, in growing and in mature root hairs were performed by time-lapse visualization of late endosomes with GFP-2xFYVE marker, and subsequently evaluated by ImageJ software. Using the semi-automatic approach, individual, dancing-, and clustered endosomes were recognized to be more abundant in bulge stage and growing root hairs compared to mature root hairs (Figures 7A–C). In general, the endosomal population in the bulge stage of root hair emergence and in growing root hairs was significantly larger than in mature root hairs (Figure 7D). However, the number of clustering and dancing events per second was higher in growing root hairs than in emerging and mature root hairs (Figure 7E). For all root hair developmental stages, dancing-endosomes occurred in higher frequencies than the clustered ones (Figure 7E).

**Figure 8:**
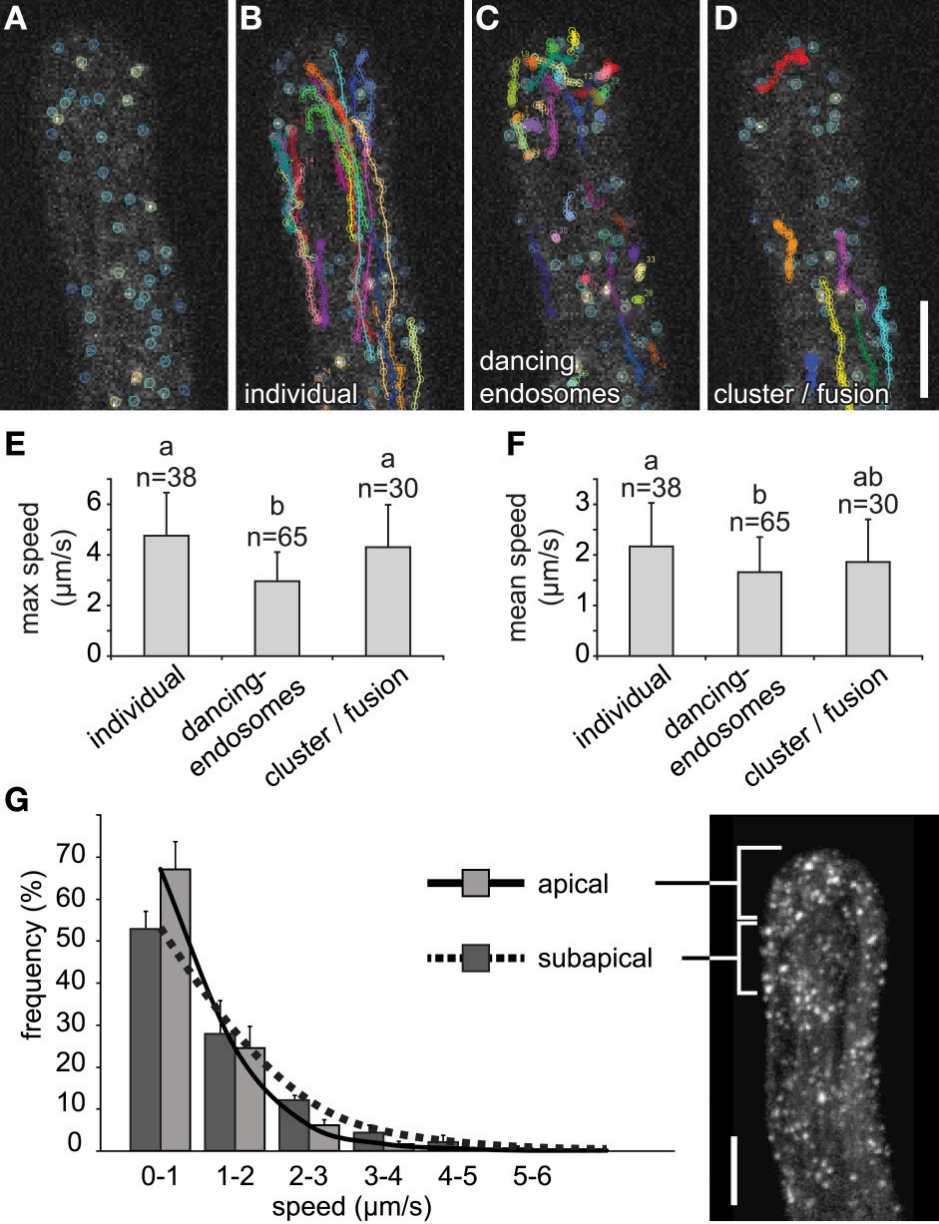
**Semiautomatic identification and quantification of individual, dancing-endosomes, and clustered/fused late endosomes visualized by GFP-2xFYVE**. **(A)** Color-coded marking of late endosomes in growing root hair based on total fluorescence intensity. **(B–D)** Semiautomatic detection and trajectory tracking of individual late endosomes **(B)**, dancing-endosomes **(C)**, and clustered/fused late endosomes **(D)** in growing root hair. All images correspond to one z-frame selected from acquired xyzt-series, color coding showing the accumulative changes of detected interactions and trajectories after 12 **(A)**, 49.5 **(B)**, and 50 **(C,D)** s of recording. **(E,F)** Maximum speed **(E)** and mean speed **(F)** estimation of individual, dancing-endosomes, and clustered/fused endosomes in growing root hairs. More than 30 endosomes per event from 10 growing root hairs were used for speed estimations. **(G)** Distribution of late endosomes according to their speed were measured in the apical (0–10 μm below the tip) and the subapical (10–20 μm below the tip) zones of five growing root hairs. Error bars represent SD. Different letters above the bars indicate significant difference [Wilcoxon rank sum test with Holm's correction (*p* < 0.01)]. In **(G)** *n* = 5 root hairs of individual plants with total of 22,278 measurements. Scale bars represent 10 μm.

All of these endosomal interaction events were recognized and followed visually using color-coded scaling corresponding to total fluorescence intensity (Figures 8A–D). The individual endosomes that did not interact by dancing or clustering/fusion, traveled for long distances and were found preferentially in subapical zones (Figure 8B). Dancing-endosomes were preferentially located in the root hair tip and traveled only short distances (Figure 8C), while clustered endosomes were mainly located in subapical zone (Figure 8D). The maximal speed of dancing-endosomes was significantly reduced in comparison to individual and clustered/fused endosomes (Figure 8E). The clustered/fused endosomes did not move with altered maximal speed, however, they showed discontinuous movements. Consequently, the mean speed was reduced, with respect to individual endosomes, while it was comparable with the mean speed of dancing-endosomes (Figure 8F). Importantly, carefully selected line with GFP-2 × FYVE marker displayed no artificial effects of root hair morphology or root hair tip growth. Root hair growth rate of GFP-2 × FYVE line (1.941 ± 0.362 μm/min, SD) was very similar to that of control Col-0 plants (1.954 ± 0.354 μm/min, SD) and root hairs were morphologically undistinguishable in both lines.

A significant difference in the speed between endosomal subpopulations in apical and subapical regions of the growing root hair was revealed also by automatic speed estimation using DiaTrack software (Supplementary Tables S3, S4; Supplementary Figures S1E,F). In the apical region of root hairs, slower movements were determined as compared to those in the subapical region (Figure 8G). The lower speed of the endosomes located in the apical region supports the higher frequency of dancing-endosomes in this zone.

## Discussion

In this study, we characterized the localization, dynamic properties, and interactions of endosomes during root hair growth at high temporal and spatial resolution by live imaging using spinning disc microscopy. We observed distinct organization and motile behavior of early and late endosomes related to particular root hair domains and developmental stages. A new transient interaction between late endosomes was identified and was accordingly termed dancing-endosomes. This study demonstrates the power, high accuracy, and improved spatio-temporal resolution of spinning disc microscopy combined with post-acquisition multitracking analyses using DiaTrack software and semiautomatic tools for quantitative characterization of endosomes in developmental process of root hair formation.

### Global analysis of size and intracellular distribution of endosomes during root hair development

Early and late endosomes were followed using well characterized fluorescently-tagged molecular markers that localize to or bind to these organelles. Thus, early endosomes were visualized by YFP-VTI12 and GFP-RabA1d markers (Sanderfoot et al., 2001; Uemura et al., 2004; Ovečka et al., 2010; Berson et al., 2014), while late endosomes were visualized by YFP-RabF2a, YFP-RabF2b, and GFP-2xFYVE markers (Gillooly et al., 2001; Ueda et al., 2004; Voigt et al., 2005a; Vermeer et al., 2006, 2009; Haas et al., 2007; Geldner et al., 2009). Similarly to biosensors designed according to phosphatidylinositol phosphate species specificity (Vermeer et al., 2009; Simon et al., 2014), the use of known fluorescently-tagged proteins in the identification of early and late endosomes have enabled the optimization of techniques of live microscopy and image analysis of endosomal behavior.

During root hair initiation, growth, and maturation, endosomes showed different intracellular distributions, consistent to membrane trafficking requirements related to root hair developmental stages. Early endosomes visualized with GFP-RabA1d and YFP-VTI12 markers accumulated in the tip of emerging and growing root hairs, while the late endosomes marked by GFP-2xFYVE showed only a partial accumulation at the tips during bulge formation in differentiating root trichoblasts. In later developmental stages of root hair elongation, late endosomes visualized by GFP-2xFYVE did not show any specific localization. Vesicular trafficking is essential during root hair site selection, determination, and subsequent hair outgrowth (Richter et al., 2011). It is modulated by several structural changes of the actin cytoskeleton (Ringli et al., 2002), cell wall composition (Park et al., 2011), and by accumulation of structural sterols in the plasma membrane (Ovečka et al., 2010). Early endosomes visualized by GFP-RabA1d and YFP-VTI12 markers showed higher area fraction (cell occupation) than late endosomes. They preferentially accumulated in the vesicle-rich apical dome of growing root hairs, similarly to compartments early-stained by FM4-64 (Ovečka et al., 2010; Berson et al., 2014). Tip growth requires an adequate balance of macromolecular supply, retrieval, and recycling (Cole and Fowler, 2006; Campanoni and Blatt, 2007) and consistently, it can be delayed by disruption of vesicle trafficking with brefeldin A (BFA; Richter et al., 2011). Thus, early endosomes/TGN compartments play an important role during polar growth, mainly because they represent a convergence point of endocytotic and secretory trafficking pathways (Dettmer et al., 2006; Berson et al., 2014). After maturation from early endosome/TGN, the late endosomes are mainly responsible for vesicular transport to the vacuole (Bottanelli et al., 2011; Contento and Bassham, 2012).

The distribution of early and late endosomes in growing root hairs is clearly related to membrane trafficking activity which is precisely regulated during ongoing cell developmental program. The area fraction occupied by early and late endosomes was higher in growing than in mature root hairs. Consistently with this, no obvious accumulation of endosomal compartments in matured root hairs was observed after finishing their tip growth (Voigt et al., 2005a; Berson et al., 2014).

The identification and measurement of endosomal populations by automatic DiaTrack analysis revealed that early endosomes visualized by GFP-RabA1d and YFP-VTI12 markers showed smaller diameter than late endosomes, labeled by YFP-RabF2a and YFP-RabF2b markers. The most frequent size of early endosomes (around 180 nm) corresponded very well to previous reports (150–200 nm) based on transmission electron microscopy (Hause et al., 2006; Lam et al., 2007; Müller et al., 2007). The difference in the frequency distribution of GFP-RabA1d and YFP-VTI12 seems to be consistent with their localization to two TGN subpopulations. While RabA1d is likely associated with rapid TGN trafficking (Berson et al., 2014), VTI12 is present in TGN and partially also in the pre-vacuolar compartment (Uemura et al., 2004). The exact size of an object depends not only on the spatial resolution of the optical system, but also on the signal-to-noise ratio and the algorithms used for the measurement. In any case, size measurements in fluorescence microscopy cannot give exact values for small objects close to the diffraction limit. The real size range of early endosomes was well corroborated by means of SIM live imaging. Similarly, the highest proportion of late endosomes showed a typical size (around 330 nm), which also corresponds very well to previous transmission electron microscopy studies (200–500 nm; Hause et al., 2006). By using SIM imaging, again we have found a similar average size of GFP-2xFYVE and YFP-RabF2a labeled late endosomes. The above results suggest that the DiaTrack built-in algorithm reconstructs endosomes to their near physical size. The irregular size of the individual endosomal compartments is consistent with endosome maturation concept. The size of endosomes increased as a result of clustering or putative fusion events and subsequent maturation, which has been clearly observed in the path from early to late endosomes (Ueda et al., 2004; Hause et al., 2006). Recent study also revealed a relationship between vesicle size of FYVE-positive endosomes and PI3P content (Puchner et al., 2013). Thus, PI3P binding sites on the vesicle surface correlated with size increase during endosome maturation based on putative vesicle fusion.

### Dynamics of endosomal compartments in root hairs

Early endosomes showed more constant, continuous and faster movements than late endosomes which exhibited discontinuous movements, frequent changes in speed and slower mobility. These changes were registered by kymographs, which have been a useful tool to characterize the endosomal behavior, mainly to dissect spatial motility, e.g., anterograde and retrograde mobility and its relationship with motor proteins (Schuster et al., 2011; Egan et al., 2012). The dynamic behavior of early and late endosomes was clearly differentiated in two mobility patterns: continuous and discontinuous movements. Thus, early and late endosomes in growing root hairs showed significant differences in the pattern and speed of their movements. In filamentous hyphae of fungi, early endosomes showed faster velocity than mature endosomal structures (Egan et al., 2012). Similarly in Hela cells, early endosomes followed by GFP-Rab5 moved faster than lysosomes (Flores-Rodriguez et al., 2011). Thus, the differential speed and behavior of early endosomes compared with late endosomes might be related to endosomal maturation, which includes interaction and fusion events.

### Dancing endosomes and synchronized movements

Late endosomes visualized with GFP-2xFYVE marker moved frequently together in a synchronized manner for a certain period of time. Such late endosomes approached each other, closely interacted for short time. Subsequently, they might keep a relatively constant distance for another period of time before quick separation. We observed the same behavior also in late endosomes visualized with YFP-RabF2a marker and we term this movement pattern dancing-endosome interaction. Similarly to other transient interactions such as kiss-and-run, the physical interaction may allow a content interchange between two structures; however, kiss-and-run has been previously reported to play a role mainly during vesicle recycling in mammalian cells. Kiss-and-run interactions occur in early recycling pathway of synaptic vesicles which rapidly release their content at the active zone by a transient bridge (Ryan and Reuter, 2001). The fusion pore remains open only transiently, the secretory vesicle content is discharged and after, a rapid endocytosis undergo at the same location (Ryan and Reuter, 2001; Rizzoli and Jahn, 2007). Several lines of evidence support this mode of partial membrane fusion and retrieval without the full collapse of the vesicle into the plasma membrane (Alabi and Tsien, 2013). Size and pH-dependent photoluminescence changes define kiss-and-run from full-collapse fusion (Zhang et al., 2009) and contrary to dancing-endosomes, the fusion pore open time is less than 1 s during kiss-and-run interaction (Gandhi and Stevens, 2003; Zhang et al., 2009). Although, kiss-and-run interactions were occasionally observed between early and late endosomes during lysosome biogenesis (Duclos et al., 2003) and between late endosomes (Vermeer et al., 2006), the time for this transient fusion and retrieval as well as its dynamics have not been well described. Therefore, we propose dancing-endosomes as a new transient interaction between late endosomes in plants. This type of interaction can be involved in endosomal maturation or eventually in the recycling from late endosomes/multivesicular bodies to the TGN. Temporal changes in distances between dancing endosomes might suggest that it is regulated by putative stepped tethering. Although the specific role of such interactions needs to be clarified more precisely in the future experiments, the previous studies provided evidence suggesting transport from prevacuolar compartments/MVBs to the Golgi (Pfeffer, 2001; daSilva et al., 2005, 2006), supported also by the close proximity observed between YFP–2xFYVE-labeled vesicles and STtmd–CFP-labeled Golgi stacks (Vermeer et al., 2006). Thus, these interactions might be involved in selective endocytotic content sorting destined either for degradation (Duclos et al., 2003) or recycling.

Dancing endosomes were recognized in quantitative live cell imaging by their proximity (less than 1 μm) and temporal intensity increase. This type of interaction was more frequent during a brief period of time (more than 1 s) and such endosomes moved together over short distances. More stable increase in the fluorescence intensity and its longer duration suggested clustering and/or fusion events. Consistently, during maturation of endosomal compartments, clustering, and putative fusion events increased compartment size and fluorescence intensity. It was established quantitatively using live imaging analysis of endosomes visualized by eGFP-Rab5 (Helmuth et al., 2009) and recently, a new approach for molecules counting in single organelles demonstrate the correlation during endosomal maturation between size and fluorescence saturating levels of PI3P (Puchner et al., 2013). Thus, an increase in the fluorescence intensity of endosomes labeled by GFP-2xFYVE is consistent with clustering or putative fusion events, especially because FYVE domain marker binds specifically to PI3P in the membranes of late endosomal compartments (Gillooly et al., 2001). Fusion events between endosomes can be induced also by wortmannin treatment, which promotes both homotypic and heterotypic fusions of MVBs and early endosomes (Wang et al., 2009; Takáč et al., 2012). Wortmannin appears to block protein recycling from late endosomes/MVBs to TGN (daSilva et al., 2005, 2006), thus it induces enlargement of MVBs (Vermeer et al., 2006; Wang et al., 2009) and heterotypic fusions of TGN and MVB (Takáč et al., 2012). Similar structures have been induced by reduction of PI3P using PI3K-specific inhibitor LY294002 (Takáč et al., 2013), which also affected root hair growth (Lee et al., 2008).

### Spatial regulation of endosomal interactions in root hairs

Color-coded scale depicting the total fluorescence intensity helped to recognize and follow individual, dancing-endosomes, and clustered/fused endosomes. The fluorescence intensity of clustered/fused endosomes was significantly higher than in individual and dancing-endosomes. This is consistent with the morphological changes in the endosomal structures (Helmuth et al., 2009; Puchner et al., 2013), and with their distribution and dynamics during root hair development. The detection of individual, dancing-endosomes, and clustered/fused endosomes within the entire population was better in growing root hairs than in bulges and mature root hairs. Dancing-endosomes were observed in higher frequency of events than clustering/fusions. Since tip growth requires a balance of macromolecular supply, retrieval and recycling (Cole and Fowler, 2006; Campanoni and Blatt, 2007), part of this balance is established by TGN/early endosomes and late endosomes during secretory and endocytotic trafficking (Dettmer et al., 2006; Berson et al., 2014) and during vesicular transport to the vacuole (Bottanelli et al., 2011; Contento and Bassham, 2012), respectively. Here we show that in growing root hairs, individual endosomes located in subapical zones traveled long distances and did not interact or cluster/fuse. On the other hand, endosomes preferentially located in the root hair tip interacted as dancing-endosomes and traveled short distances during this interaction. This root hair tip zone is depleted of thick actin cables but it is enriched with dense meshwork of short actin microfilaments which appears to be crucial for root hair growth and development (Baluska et al., 2000; Voigt et al., 2005b). Moreover, both endosomal organization and dynamics are closely related to the actin cytoskeleton and its pharmacological impairment affects both endosomal morphology and motility in root hairs (Voigt et al., 2005a). Thus, short and highly dynamic actin microfilaments at the root hair tip influence endosomal movements and possibly also their interaction and fusion events. Endosomal behavior during dancing interaction as well as tip-localization of this interaction suggest that it is an effective strategy for selective sorting of endocytotic content destined either for degradation in the vacuole (Duclos et al., 2003) or recycling to the TGN (Pfeffer, 2001; daSilva et al., 2005, 2006) during tip growth. The maximal speed of dancing-endosomes was significantly reduced compared to individual and clustered/fused endosomes. Finally, clustered/fused endosomes were mainly located in subapical root hair zones.

**Figure 9:**
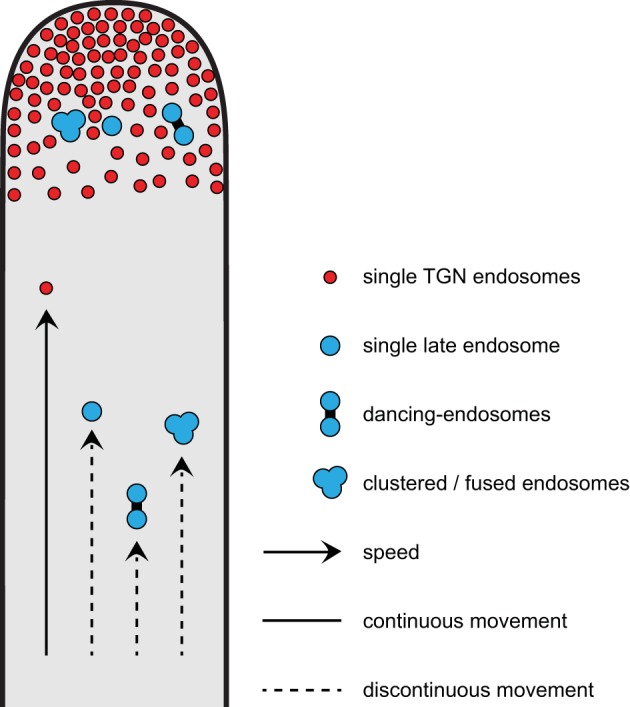
**Model depicting different modes of movements for early and for late endosomes in growing root hairs**. Early endosomes enriched in the growing tip show faster movements than late endosomes. For late endosomes, single endosomes move faster than clustered/fused ones, but dancing-endosomes show the slowest movement.

A model summarizing different modes of movements for early and late endosomes (single, dancing-endosomes, and clustered/fused endosomes) in growing root hairs is presented in Figure 9. Early endosomes are enriched in the root hair tip and show faster movements at constant velocities as compared to late endosomes. Late endosomes show discontinuous movement patterns. The movements of dancing late endosomes are significantly slower than those of single late endosomes (Figure 9).

## Conclusions

Differential organization, motile behavior, and interactions of endosomes were related to particular root hair zones and developmental stages of root hair growth. Early endosomes showed mainly continuous movements while late endosomes mostly moved discontinuously. Late endosomes occasionally moved together in a synchronized manner showing a transient dancing interaction (dancing endosomes) that was not described before in eukaryotic cells. Time-lapse live microscopy coupled with single automatic particle analysis becomes a new quantitative approach to study organization and mobility patterns of early and late endosomes, and to recognize interactions of late endosomes in plant cells.

## Author contributions

DW performed spinning disc microscopy bioimaging and automatic image analysis using DiaTrack and ImageJ. AR performed semiautomatic image analysis of endosomes and dynamic evaluation using ImageJ. GK performed SIM studies. OŠ and MO prepared biological materials for this study. JŠ designed and coordinated all experiments. DW, AR, GK, and JŠ performed the data analysis. DW, AR, BV, GK, and JŠ wrote the manuscript. All authors read and approved the final manuscript.

## Funding

This work was supported by National Program for Sustainability I (grant no. LO1204) provided by the Czech Ministry of Education and by Institutional Fund of Palacký University Olomouc (GK and OŠ).

### Conflict of interest statement

The authors declare that the research was conducted in the absence of any commercial or financial relationships that could be construed as a potential conflict of interest.

## Supplementary material

Supplementary Table S1 **Population of endosomal compartments used for diameter measurements**.

Supplementary Table S2 **Population of endosomal compartments and distribution frequencies (in percentage) of continuous vs. discontinuous movements**.

Supplementary Table S3 **Distribution of endosomal population from apical region of root hairs according to speed**.

Supplementary Table S4 **Distribution of endosomal population from subapical region of root hairs according to speed**.

Supplementary Movie S1 **Dynamics of early and late endosomes visualized by fluorescent markers in growing root hairs**.

Supplementary Movie S2 **Stop-and-go movement of late endosomes visualized by GFP-2xFYVE**.

Supplementary Movie S3 **Dancing interaction and synchronized movements of late endosomes visualized by GFP-2xFYVE**.

Supplementary Movie S4 **Dancing interaction and synchronized movements of the same endosomes shown in Movie S3 (visualized by GFP-2xFYVE)**. The tracking data was used to translate the movie around the position of one of the endosomes using Adobe After Effects.

Supplementary Movie S5 **Dancing interaction and synchronized movements of late endosomes visualized by YFP-RabF2a**.

Supplementary Movie S6 **Late endosomes visualized by GFP-2xFYVE undergoing clustering/fusion**. Three endosomes marked by different arrows approximate to each other and sequentially cluster/fuse together producing large endosome.

Supplementary Figure S1 **Quantitative characterization of early and late endosomes using automatic and semiautomatic analysis**. Detection and diameter estimation of endosomal compartments visualized by GFP-RabA1d **(A)** and YFP-RabF2a **(B)** markers using DiaTrack software. Maximal speed determination of endosomes visualized by GFP-2xFYVE **(C)** and GFP-RabA1d **(D)** markers from kymographs generated in ImageJ. Automatic identification **(E)** and trajectory tracking **(F)** of late endosomes visualized by GFP-2xFYVE marker using DiaTrack software.
