# Supplementary material for: Endosomal Interactions during Root Hair Growth
Source: Front Plant Sci. 2016 Jan 29;6:1262. doi: 10.3389/fpls.2015.01262 (PMC4731515; doi:10.3389/fpls.2015.01262)
Supplement: Supplementary Table S1 — Population of endosomal compartments used for diameter measurements. [file Table1.PDF]

Supplementary Table S1 Population of endosomal compartments used for diameter measurements.

Fluorescent marker	GFP-RabA1d	YFP-VTI12	YFP-RabF2a	YFP-RabF2b
Number of measurements	67126	9824	6513	5947

Supplementary Table S2 Population of endosomal compartments and distribution frequencies (in percentage) of continuous versus discontinuous movements.

fluorescent	GFP-RabA1d	YFP-VTI12	YFP-RabF2a	YFP-RabF2b	GFP-FYVE
marker	(n = 673)	(n = 628)	(n = 651)	(n = 495)	(n = 609)
continuous	80,2	54,3	39,3	38,0	39,6
discontinuous	19,8	45,7	60,7	62,0	60,4

Supplementary Table S3 Distribution of endosomal population from root hair apical region according to speed.

	Distribution by percentage in root hair nr. 1-5					
speed	1	2	3	4	5	average
[µm/s]	(n = 1434)	(n = 3718)	(n = 2107)	(n = 3674)	(n = 1969)	(n = 12902)
0-1	60,67	61,81	66,92	69,87	77,09	67,27 ± 6,65
1-2	28,66	29,88	25,82	20,79	17,83	24,60 ± 5,15
2-3	7,11	7,42	5,32	6,48	4,06	6,08 ± 1,39
3-4	2,58	0,89	1,33	2,15	0,86	1,56 ± 0,77
4-5	0,84	0,00	0,62	0,60	0,15	0,44 ± 0,35
5-6	0,14	0,00	0,00	0,08	0,00	0,04 ± 0,06
6-7	0,00	0,00	0,00	0,00	0,00	0,00 ± 0,00

Supplementary Table S4 Distribution of endosomes population from root hair subapical region according to speed.

	Distribution by percentage in root hair nr. 1-5					
speed	1	2	3	4	5	average
[µm/s]	(n = 1373)	(n = 2966)	(n = 1620)	(n = 1822)	(n = 1595)	(n = 9376)
0-1	50,25	48,21	53,27	59,55	53,67	52,99 ± 4,30
1-2	32,34	37,39	28,27	15,92	26,14	28,01 ± 8,01
2-3	12,09	12,58	13,40	12,02	10,16	12,05 ± 1,19
3-4	3,79	1,82	3,95	6,75	5,14	4,29 ± 1,82
4-5	1,17	0,00	1,11	4,23	3,51	2,00 ± 1,78
5-6	0,36	0,00	0,00	1,26	1,25	0,58 ± 0,64
6-7	0,00	0,00	0,00	0,22	0,13	0,07 ± 0,10
