# Supplementary material for: Endosomal Interactions during Root Hair Growth
Source: Front Plant Sci. 2016 Jan 29;6:1262. doi: 10.3389/fpls.2015.01262 (PMC4731515; doi:10.3389/fpls.2015.01262)
Supplement: Supplementary Figure S1 — Quantitative characterization of early and late endosomes using automatic and semiautomatic analysis. Detection and diameter estimation of endosomal compartments visualized by GFP-RabA1d (A) and YFP-RabF2a (B) markers using DiaTrack software. Maximal speed determination of endosomes visualized by GFP-2xFYVE (C) and GFP-RabA1d (D) markers from kymographs generated in ImageJ. Automatic identification (E) and trajectory tracking (F) of late endosomes visualized by GFP-2xFYVE marker using DiaTrack software. [file Presentation1.PDF]

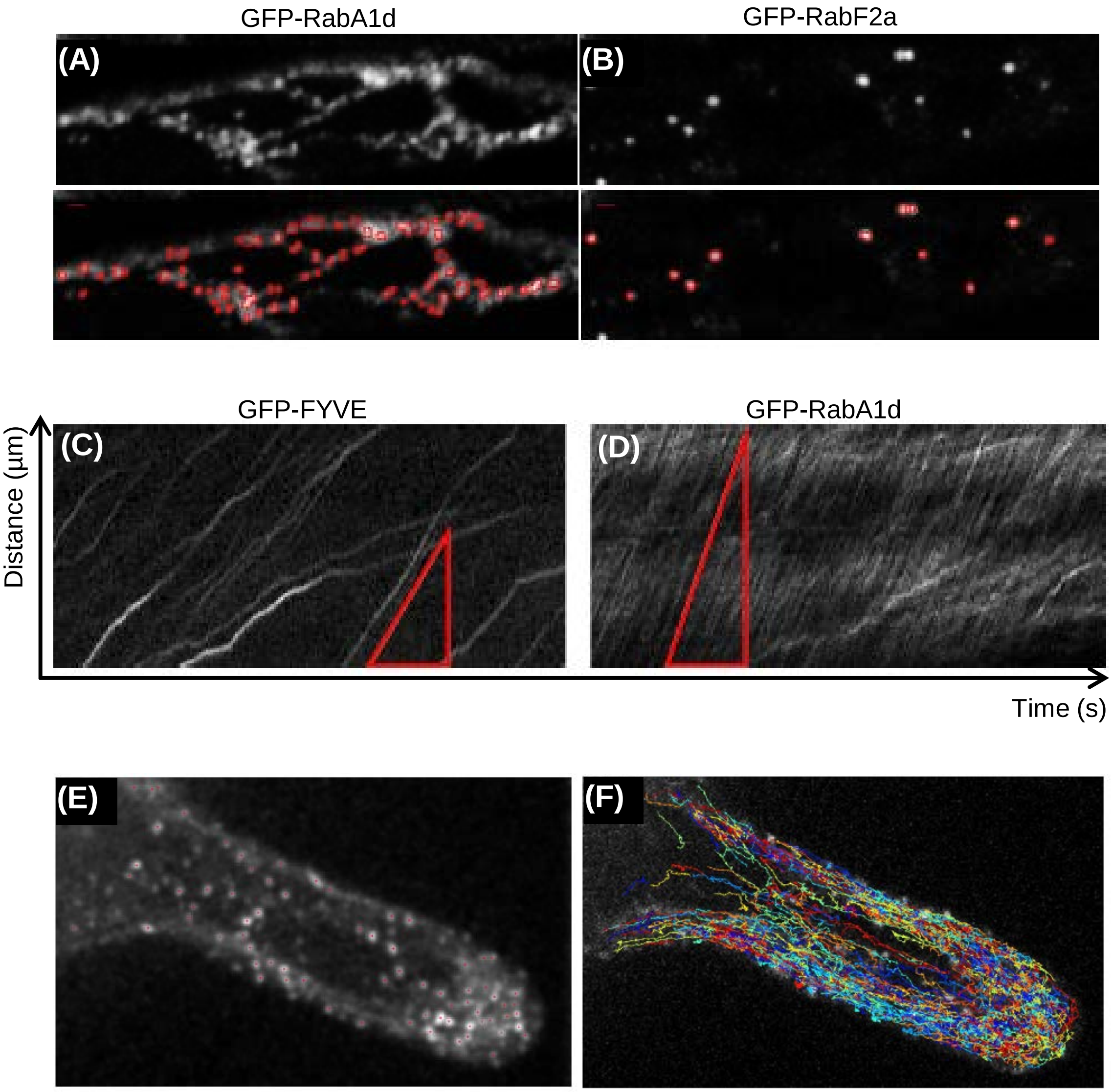

Supplementary Figure S1. Quantitative characterization of early and late endosomes using automatic and semiautomatic analysis. Detection and diameter estimation of endosomal compartments visualized by GFP-RabA1d (A) and GFP-RabF2a (B) markers using DiaTrack software. Maximal speed determination of endosomes visualized by GFP-2xFYVE (C) and GFP-RabA1d (D) markers from kymographs generated in ImageJ. Automatic identification (E) and trajectory tracking (F) of late endosomes visualized by GFP-2xFYVE marker using DiaTrack software.
